# Antitumoral efficacy of silver nanoparticles reduced with β-D-glucose on a canine transmissible venereal tumor cell line

**DOI:** 10.3389/fvets.2026.1794174

**Published:** 2026-06-08

**Authors:** Natanael Palacios Estrada, Silvia Elena Santana Krismkaya, David Reding Hernández, Kenia Arisbe Moreno Amador, Diana Ginette Zarate Triviño, Moisés Armides Franco Molina, Jorge R. Kawas, Cristina Rodríguez Padilla

**Affiliations:** 1 Laboratorio de Inmunología y Virología, Facultad de Ciencias Biológicas, Universidad Autónoma de Nuevo León, San Nicolas de los Garza, Nuevo León, Mexico; 2 Posgrado Conjunto Agronomía-Veterinaria, Universidad Autónoma de Nuevo León, Escobedo, Nuevo León, Mexico

**Keywords:** apoptosis, canine transmissible venereal tumor, CTVT, oxidative stress, reactive oxygen species, silver nanoparticles

## Abstract

**Background:**

Canine transmissible venereal tumor represents a significant threat to animal health, particularly due to the large global population of stray dogs. The standard treatment for cTVT is chemotherapy, primarily based on vincristine. However, the use of this drug is associated with a high cost of treatment, the development of tumor resistance, and the occurrence of adverse side effects. Therefore, silver nanoparticles have emerged as a potential therapeutic alternative due to their documented cytotoxic effects on various cancer cell lines. Nevertheless, their cytotoxic activity against cTVT has not yet been demonstrated.

**Methods:**

In this preliminary study, we evaluated the cytotoxic effects of silver nanoparticles reduced with β-D-glucose on a cTVT cell line. The nanoparticles were characterized by UV–vis spectrophotometry by analyzing absorbance spectra from 200 to 600 nm over a period of 27 days. Cytotoxicity was assessed using the Alamar Blue assay. Reactive oxygen species (ROS) production was measured using a ROS/RNS Detection Kit. Apoptosis was evaluated by immunocytochemical detection of caspase-3 and GAPDH, as well as by fluorescence microscopy using acridine orange/ethidium bromide (AO/EB) and DAPI staining. Additionally, blood samples from three healthy household dogs were collected to evaluate TNF-α production and cytotoxic effects on peripheral blood mononuclear cells (PBMCs). Statistical analyses were performed using one-way ANOVA with GraphPad Prism 6.

**Results:**

Silver nanoparticles significantly reduced cell viability in a dose-dependent manner. Treatment affected GAPDH expression as evaluated by immunocytochemistry, increased ROS production, and induced apoptosis, as evidenced by AO/EB and DAPI fluorescence microscopy and caspase-3 expression. Furthermore, cTVT-conditioned medium did not affect plasma TNF-α levels, as determined by ELISA. In addition, no lactate dehydrogenase (LDH) release was observed in PBMC co-culture assays, indicating an absence of cytotoxic effects on non-tumoral cells.

**Conclusion:**

Silver nanoparticles reduced with β-D-glucose induce cell death in cTVT cells without triggering an immunogenic response. These findings suggest that this nanomaterial may represent a promising novel therapeutic approach in veterinary oncology.

## 1 Introduction

Canine transmissible venereal tumor (cTVT) is a common cause of death in dogs. This neoplasia is spontaneously transmitted through sexual or mechanical contact. cTVT is the only type of cancer that can be transmitted between individuals of the same species, surpassing the barrier of the major histocompatibility complex. It can also be transmitted to members of the same family, such as wolves, foxes, and coyotes (1). The causal agent of cTVT is the tumor cell itself. During sexual or physical contact, these cells are transferred from one animal to another and invade the new host through mucous membranes or sites where the integrity of the dermis has been compromised, such as wounds or abrasions (2).

There are different treatment modalities for cTVT, including surgery, radiation therapy, and chemotherapy (1). Among these, chemotherapy is the most effective and practical treatment available. Chemotherapeutic agents such as cyclophosphamide, methotrexate, vincristine, vinblastine, and doxorubicin are therapeutically effective. The most used chemotherapy is vincristine sulfate (3), nevertheless recurrence is observed when chemotherapy is not used correctly (4). Conventional treatment in many cases cannot be provided by pet owners due to the high cost (5). Together with this and the scarcity of *in vitro* and clinical studies against cTVT, the need to develop new strategies against cTVT is necessary.

Nanoparticles have gained significant relevance due to their cytotoxic effects on different cancer cell lines (6). For the synthesis of nanoparticles, three necessary components are required: a precursor, a reducing agent, and a stabilizing agent (7). Silver is one of the most used metallic precursors for the development of antitumor therapies, due to its cytotoxic properties (8). It has been have reported that silver nanoparticles using β -D-glucose as the reducing and stabilizing agent to alter cell morphology, and induce toxicity (9). Our research group has extensively characterized and reported the antitumoral efficacy of silver nanoparticles synthesized through a green chemistry approach using β-D-glucose as a reducing agent. In previous studies involving breast cancer models, silver nanoparticles reduced with β-D-glucose (AgNPs-β-D-glucose) demonstrated dose-dependent cytotoxicity and the capacity to induce immunogenic cell death (ICD), as evidenced by the exposure of calreticulin and the release of damage-associated molecular patterns (DAMPs) such as HSP70, HSP90, HMGB1, and ATP (10). Furthermore, *in vivo* evaluations in triple-negative breast cancer (TNBC) a murine models revealed that AgNPs-β-D-glucose can effectively remodel the tumor microenvironment by increasing the infiltration of memory T cells and innate effector cells, while upregulating pro-inflammatory cytokines such as TNF-α, IFN-γ and IL-6 (10). Recent evidence also supports the potential of AgNPs-β-D-glucose as a neoadjuvant therapy; intratumoral administration significantly reduced primary tumor volume and inhibited lung metastasis through the downregulation of the proliferation marker Ki67 and the induction of a robust antitumor response (36). Therefore, the present study aims to assess the cytotoxic effects of these compounds on the cTVT cell line and the ability of cTVT cells lysed with AgNPs-β-D-glucose to stimulate the *ex vivo* production of TNF-α, offering another alternative in the treatment of this disease.

## 2 Materials and methods

### 2.1 Ethical approval

The animal study was approved by the Comité de Ética de Investigación y Bienestar Animal (CEIBA), FCB, UANL. The study was conducted in accordance with the local legislation and institutional requirements.

### 2.2 Cell culture

The cTVT cell line used was obtained from the cell line bank of the Immunology and Virology Laboratory at the Faculty of Biological Sciences of UANL. To ensure methodological transparency and reproducibility, the cTVT cell line was authenticated by both cytogenetic and molecular analyses. Identity was confirmed through karyotype analysis, revealing the pathognomonic chromosomal reduction (56, XX) and complex structural rearrangements characteristic of this lineage. Additionally, molecular validation was performed via PCR to detect the specific LINE-c-myc insertion signature. Detailed authentication reports, including metaphase images and electrophoresis gels, are provided in previous publications (11). The cells were cultured at 37 °C in an atmosphere with 5% CO_2_ and a relative humidity of 80%. For cell maintenance, Dulbecco’s Modified Eagle Medium (DMEM) cell culture medium (GIBCO® by Life Technologies™, United States) supplemented with 10% (v/v) Fetal Bovine Serum was used.

### 2.3 Synthesis of silver nanoparticles reduced with β-D-glucose

The AgNPs-β-D-glucose were synthesized and characterized as previously described by our research group (Felix et al., 2023). In brief, 10 mL of aqueous solution of β-D-glucose at a concentration of 0.3 M in a beaker was exposed to a water bath in glycerol at 120 °C for 5 min. Subsequently, 100 μL of AgNO_3_ solution was added dropwise. A total of 2.5 mM and 10 μL of 0.1 M NaOH solution were added until the color changed to yellow, which is indicative of AgNPs-β-D-glucose formation (Felix et al., 2023). In all preparations, deionized water provided by a comprehensive Milli-Q water purification solution system was used (Merck Millipore, Billerica, MA, United States).

### 2.4 Characterization of AgNPs-β-D-glucose

The synthesized nanoparticles were characterized by UV–visible spectroscopy using the NanoDrop 2000C equipment (Thermo Scientific®, United States). The spectrum was analyzed from 200 to 700 nm, and the interval of 300 to 600 nm was plotted. Size, polydispersity index (PDI) and zeta potential values were taken in a Zetasizer Nano ZS90 (Malvern Instruments, Malvern, United Kingdom) (Felix et al., 2023).

### 2.5 Cytotoxic effect

cTVT cells were seeded in a 96-well flat-bottom plate (Corning Inc. Costar®, United States) at a density of 5 × 10^3^ cells per well in 100 μL of Dulbecco’s modified Eagle’s medium (DMEM) supplemented with 5% Fetal Bovine Serum (FBS) culture medium. The cells were incubated for 24 h at 37 °C in an atmosphere of 5% CO_2_ and 80% relative humidity. They were then treated with different concentrations of AgNPs-G (1–100 μM) for 24 h. After the treatment period, the cells were washed with 1X phosphate-buffered saline (PBS). Then, 100 μL of Alamar Blue (Sigma, St. Louis, MO, United States) at 20% *v/v* was added and incubated for 4 h under the conditions previously described. The fluorescence readings were obtained using a Synergy HT™ spectrophotometer with excitation at 535 nm and emission at 590 nm (Felix et al., 2022).

### 2.6 Detection of reactive oxygen species (ROS) and superoxide ion

cTVT cells were seeded onto sterile glass coverslips placed in 6-well plates (Corning Inc. Costar®, United States) at a concentration of 5 × 10^5^ cells per well. The cells were incubated for 24 h to allow proper adherence. After the incubation period, treatments were applied for 24 h, including an untreated control group, an LPS treatment group (1 μg/mL), a dexamethasone treatment group (5 μM), and an AgNPs-β-D-glucose treatment group at the determined LD_50_ concentration. For modulation analysis, dexamethasone or LPS were added for an additional 2 h post-treatment. The experimental protocol followed the manufacturer’s instructions for the Cellular ROS/RNS Detection Kit (ab139473, Abcam). Briefly, the supernatant was removed, and cells were washed with 1X PBS. Then, 100 μL of the ROS/RNS 3-Plex Detection Mix was added to each well, covering the coverslips, and incubated for 30 min. Following incubation, the coverslips were mounted on microscope slides and visualized using a confocal fluorescence microscope (Olympus IX70, United States). Cells were examined at excitation/emission wavelengths of 490/520 nm for ROS detection and 550/620 nm for superoxide ion detection (12). Experiments were performed in triplicate (*n* = 3) using independent cell preparations.

### 2.7 GAPDH and caspase-3 evaluation by immunocytochemistry

cTVT cells were seeded at a concentration of 5 × 10^5^ over a coverslip in a 6-well plate as previously described. The cells were incubated for 24 h to allow proper adherence, after adherence the cells were treated with the DL_50_ from AgNPs-β-D-glucose for 24 h. After the incubation time, the cells were washed with PBS 1X. Cells in coverslips were fixed with methanol/acetone 1:1 for 10 min. After that, a permeabilization solution with 0.025% of Triton X-100 in TBS was added for 5 min. Antigen recovery was performed using a buffer solution of Na 10 mM, 0.05% Tween 20 at pH 6 for 30 min.

Normal horse serum (Vector Labs, United States) was used to block non-specific sites. The samples were incubated with the primary antibodies (Table 1) at 4 °C for 24 h. The antibodies were diluted 1:1000. After incubation with the primary antibodies, a wash with 1X PBS was made, and subsequently, an incubation period with the biotinylated pan-specific secondary antibody (Vector Labs, United States) for 10 min, followed by an incubation with streptavidin-biotin (Vector Labs, United States) for 10 min. Next, the samples were incubated with the chromogenic substrate diaminobenzidine (DAB) for 2 min. The reaction was stopped by adding distilled water. The samples were counterstained with hematoxylin (Sigma Aldrich, United States) for 30 s. Subsequently, the samples were dehydrated in a xylene-alcohol series, and each coverslip was mounted onto a microscope slide using Entellan® (Merck Millipore, DE). The determination of a positive result was evidenced by the DAB reaction (cells with brown staining). The intensity of DAB was quantified using Fiji software (ImageJ, version 2.0) through the “color deconvolution” function (13, 26).

**Table 1:** Antibodies used for immunocytochemistry.

Antibodies	PM (kDa)	Specificity	Company	Cat
CAS-3	32	h, m, r, a, c	Santa Cruz Biotechnology, Inc.	sc-56053
GAPDH (G-9)	37	h, m, r	Santa Cruz Biotechnology, Inc.	sc-365062

### 2.8 Cell viability by acridine orange and ethidium bromide

To determine cell viability, acridine orange/ethidium bromide (AO/EB) staining was performed. cTVT cells were cultured in 6-well plates at a density of 1 × 10^5^ cells per well in 2 mL of supplemented DMEM medium. Each well contained a previously sterilized coverslip. Cells were incubated for 24 h to allow adherence to the coverslip. After the incubation period, cells were treated with lethal doses (LD₅₀ and LD₁₀₀) for 24 h. Subsequently, cells were washed with 1X PBS. Cells were stained with 10 μL of AO/EB at a concentration of 100 μg/mL. Stained cells were observed using a confocal fluorescence microscope (Olympus X70, USA) at excitation/emission wavelengths of 250/605 nm for ethidium bromide and 502/525 nm for acridine orange (26).

### 2.9 Treatment of cTVT cells with specific conditioned medium

Cellular lysates were prepared from cTVT cells at different concentrations (0.5 × 10^5^, 1.5 × 10^5^, 3 × 10^5^, 1 × 10^6^, 2 × 10^6^, and 3 × 10^6^ cells) following treatment with the LD_50_ or LD_100_ of β-D-glucose–reduced silver nanoparticles (AgNPs-G). Blood samples were obtained from three healthy household dogs with no previous clinical history of cTVT. Blood was collected into K_2_-EDTA tubes (BD Vacutainer®, United States). Aliquots containing 1 mL of whole blood were incubated with 100 μL of the different concentrations of cTVT cellular lysates for 24 h at 37 °C in a humidified atmosphere containing 5% CO_2_, under constant agitation on a rotor (Barnstead Thermolyne™, United States). As controls, 100 μL of vehicle (DMEM) was used as a negative control, and 10 μg of lipopolysaccharide (LPS) derived from *Escherichia coli* O111:B4 (Sigma, United States) was used as a positive control. After incubation, samples were centrifuged at 3,500 rpm for 15 min, and plasma was collected for TNF-α determination as described below. In a parallel experiment, peripheral blood mononuclear cells (PBMCs) were isolated by density gradient centrifugation at 400×g for 30 min using Polymorphprep™ (Alere Technologies AS, Norway). PBMC viability was subsequently assessed using the Alamar Blue assay.

### 2.10 Quantification of tumor necrosis factor alpha

Reagents, standards, and plasma samples were prepared according to the manufacturer’s instructions Canine TNF-α ELISA, (ab193687, Abcam). Standards and samples were added to microplate wells pre-coated with anti–TNF-α antibodies. Subsequently, a biotinylated detection antibody was added to all wells, followed by the addition of a streptavidin solution. The reaction was developed using tetramethylbenzidine (TMB) substrate and stopped by adding 0.2 M sulfuric acid. Absorbance was immediately measured at 450 nm using a microplate reader (Synergy™ HT, United States). TNF- α concentrations were calculated using a standard curve generated by linear regression. Data represent the mean ± SEM of *n* = 3 independent experiments (14).

### 2.11 PBMC-mediated cytotoxicity assay (LDH release)

Cytotoxicity mediated by PBMCs sensitized with cellular lysates was evaluated by measuring lactate dehydrogenase (LDH) release. Cellular lysates were prepared from cTVT cells treated with the LD_50_ and LD_100_ concentrations of AgNPs-G for 24 h. These lysates were then used to sensitize PBMCs, which were isolated from canine blood by centrifugation using Polymorphoprep™ (Alere Technologies AS, NO) at 400×g for 30 min. After sensitization, PBMCs were incubated for 24 h at 37 °C in a humidified atmosphere with 5% CO_2_. Subsequently, a co-culture was performed with target cTVT cells at effector-to-target (E:T) ratios of 1:1, 1:100, and 1:1000. Cytotoxicity was determined using the CytoTox 96® Non-Radioactive Cytotoxicity Assay (Promega Corporation) following the manufacturer’s protocol. All assays were performed in triplicate (*n* = 3) using independent cell preparations (15).

### 2.12 Statistical analysis

**Figure 1:**
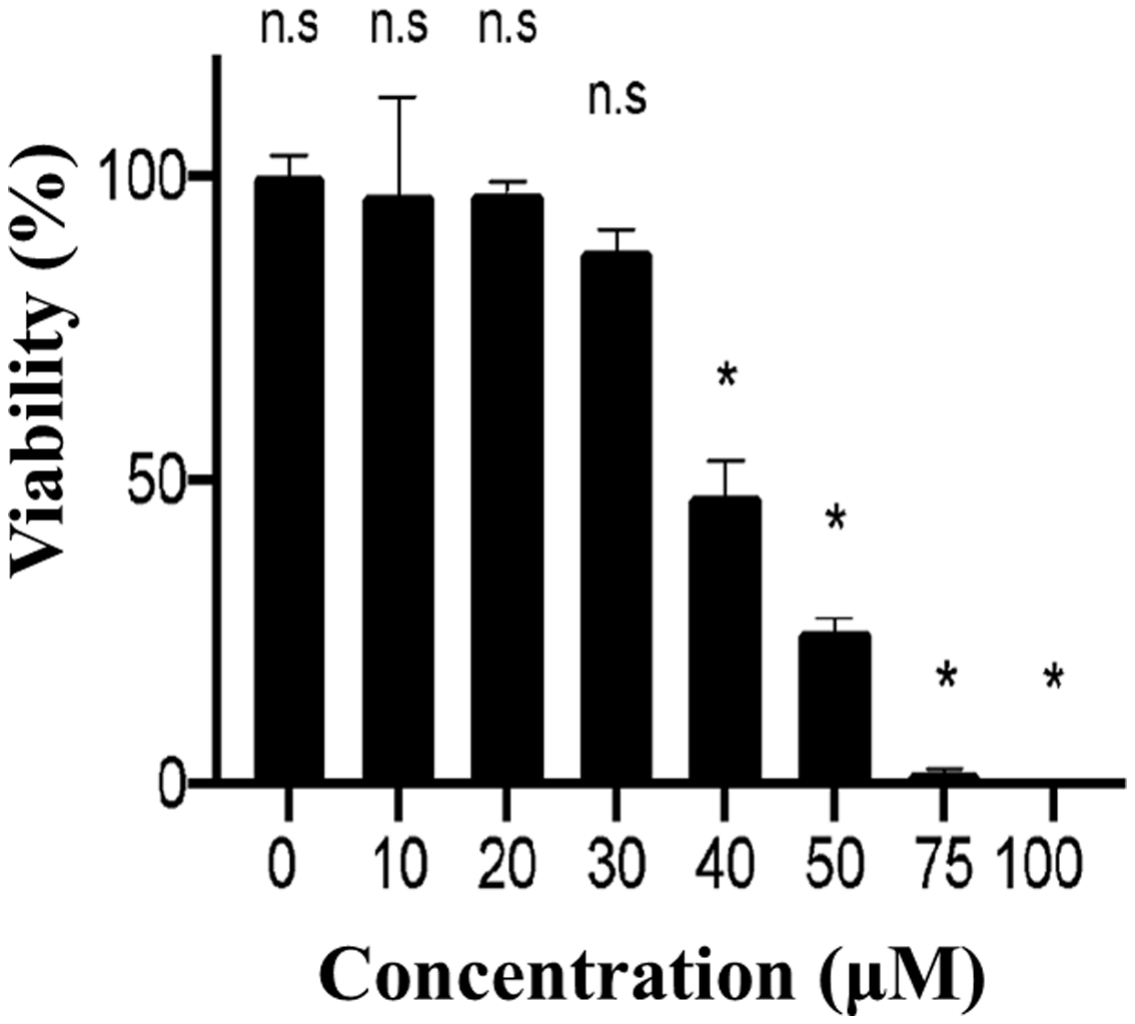
Treatment with AgNPs-G decreases cell viability. Cell viability was determined using the Alamar blue assay. Data are presented as mean ± SD. Statistical significance (*p ≤ 0.05) was determined with Dunnet’s *post hoc* test using one-way ANOVA; no significance (n.s.).

**Figure 2:**
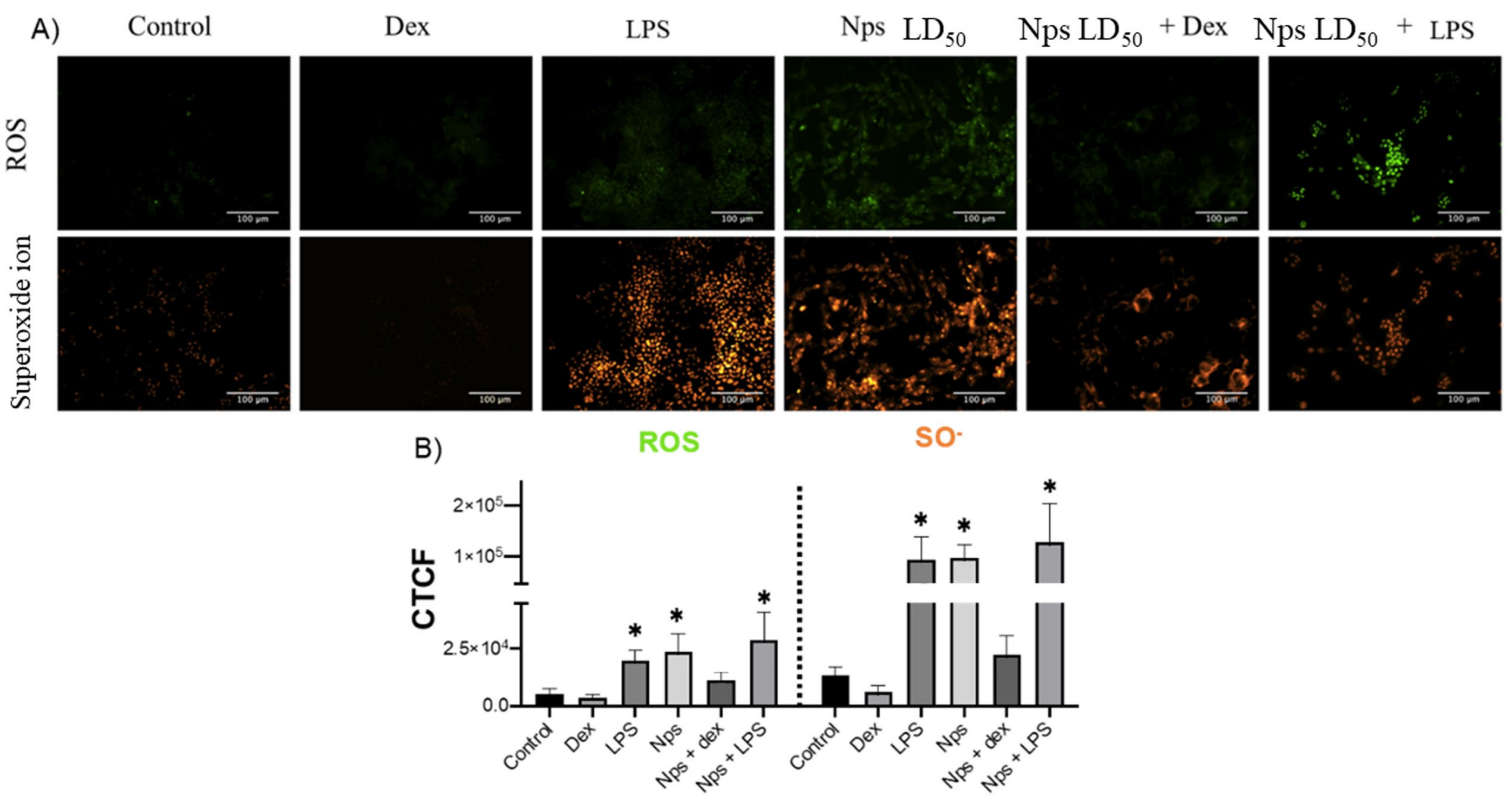
**(A)** Intracellular ROS and superoxide ion levels in cTVT cells following 24 h exposure to the LD_50_ concentration of AgNPs-G. Representative fluorescence micrographs (20× magnification) are shown. Fluorescence intensity reflects intracellular ROS and superoxide accumulation. **(B)** Quantification of corrected total cell fluorescence (CTCF). Data are presented as the mean ± SD. Statistical significance (*p < 0.05) was determined with Tukey’s post hoc test using one-way ANOVA.

**Figure 3:**
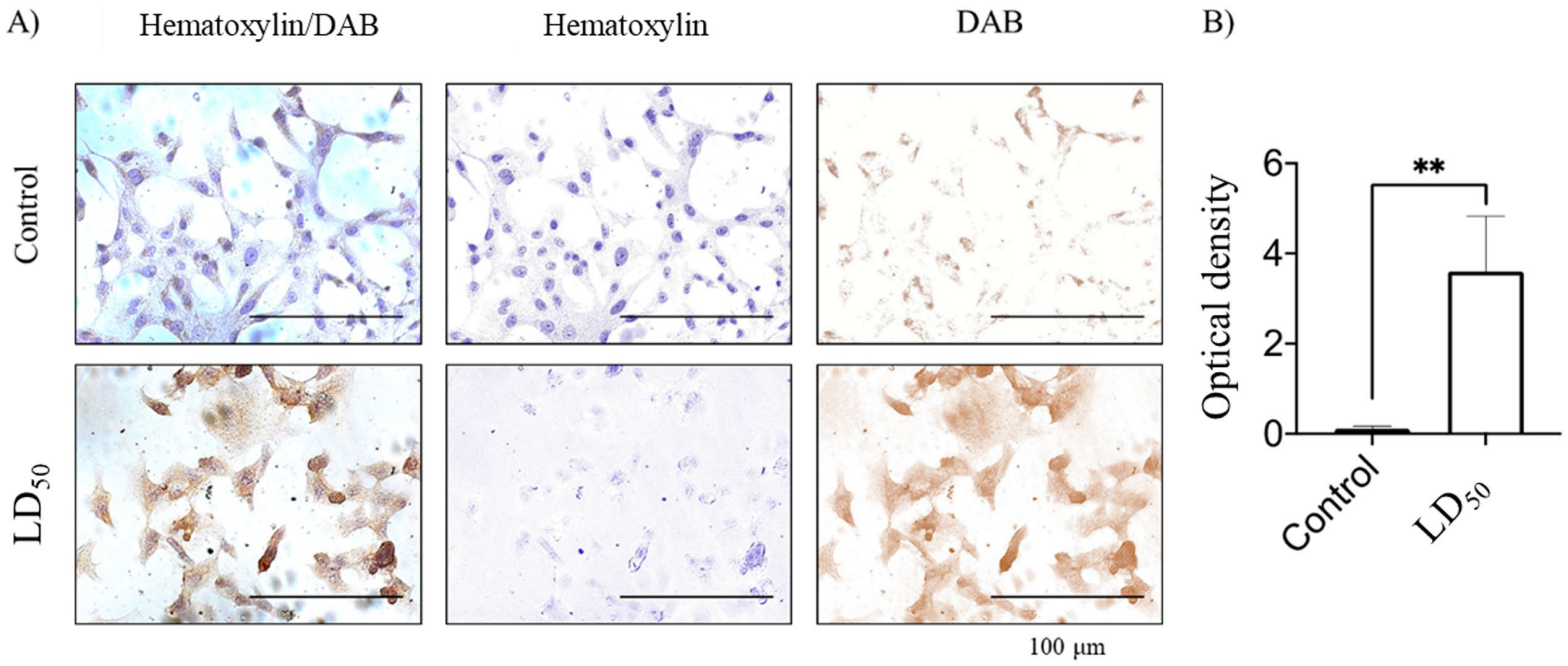
AgNPs-G treatment increases GAPDH expression in cTVT cells. **(A)** Representative immunocytochemical staining of GAPDH (DAB, hematoxylin counterstain) following 24 h exposure to the LD_50_ concentration of AgNPs-G. **(B)** Optical density analysis shows a significant increase in GAPDH levels after treatment. Data are presented as mean ± SD. Statistical significance (**p ≤ 0.01) was determined with Tukey’s post hoc test using one-way ANOVA.

**Figure 4:**
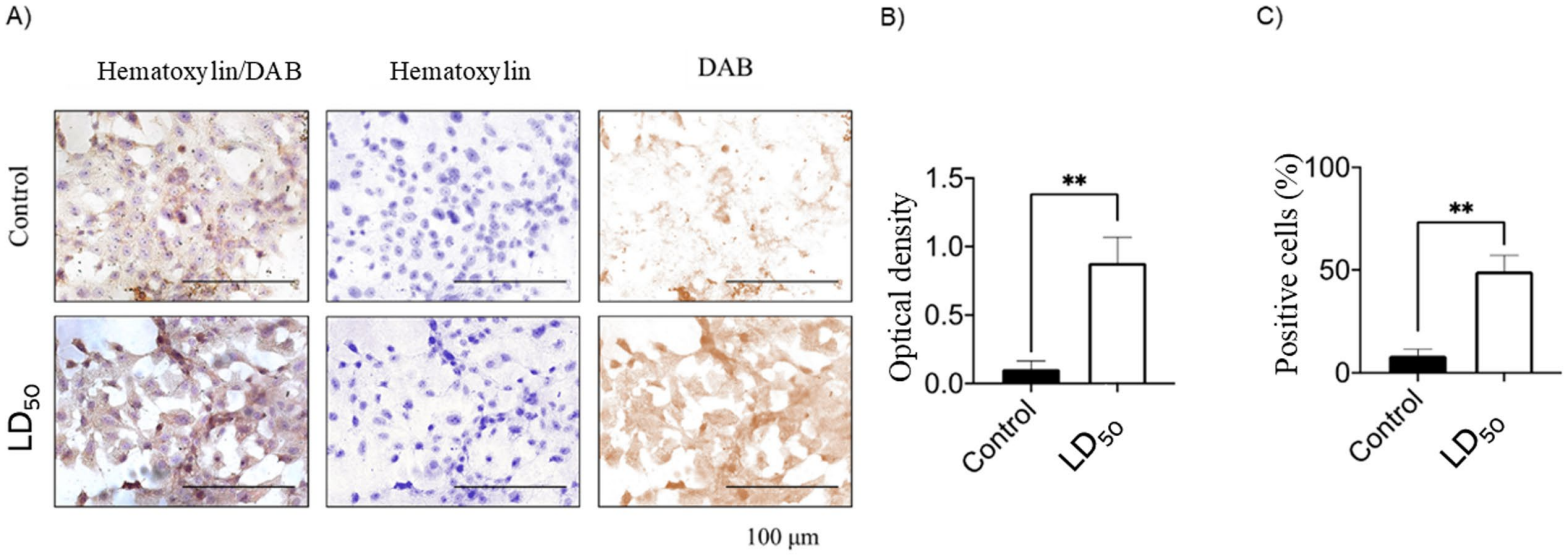
AgNPs-G treatment induces caspase-3 activation in cTVT cells. **(A)** Representative immunocytochemical staining of caspase-3 (DAB, hematoxylin counterstain) following 24 h exposure to the LD_50_ concentration of AgNPs-G. **(B)** Optical density quantification reveals a significant increase in caspase-3 expression after treatment. **(C)** Percentage of caspase-3–positive cells showing a significant elevation following AgNPs-G exposure. Data are presented as mea ± SD statistical significance (**p ≤ 0.01) determined with Dunnet’s post hoc test using one-way ANOVA.

**Figure 5:**
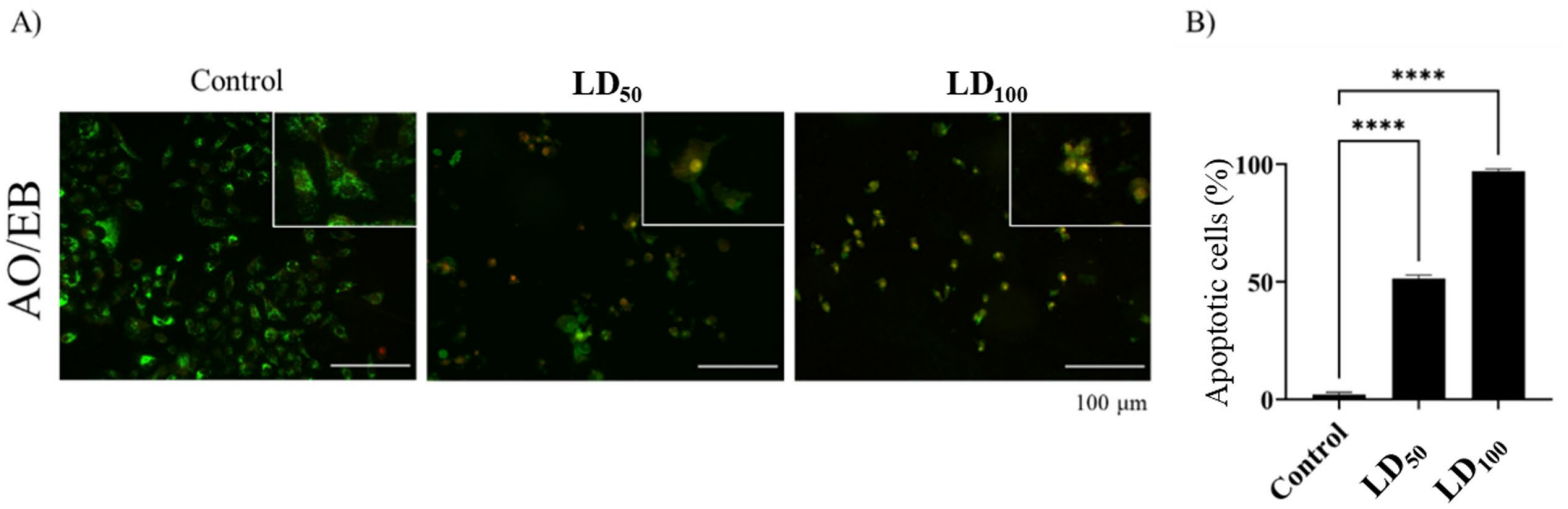
AgNPs-G induces apoptosis in cTVT cells in a dose-dependent manner. **(A)** Representative AO/EB fluorescence micrographs showing apoptotic morphology in cTVT cells following 24 h exposure to LD_50_ and LD_100_ concentrations of AgNPs-G (20× magnification). **(B)** Quantification of apoptotic cells demonstrates a significant increase after treatment. Data are presented as mean ± SD statistical significance (****p ≤ 0.0001) determined with Dunnet’s post hoc test using one-way ANOVA.

**Figure 6:**
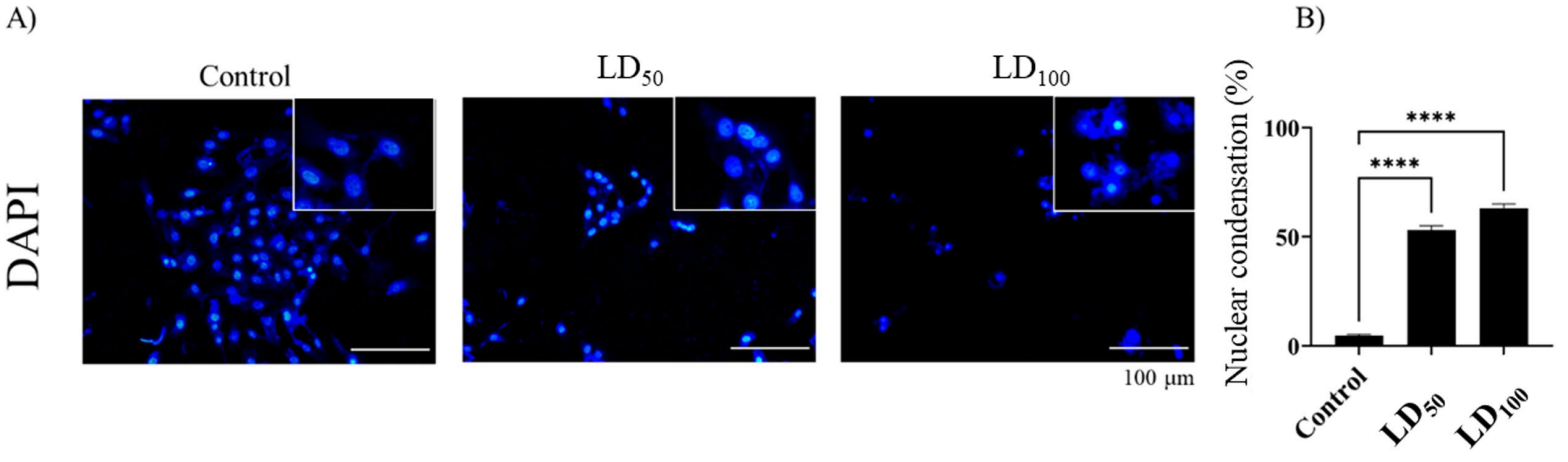
AgNPs-G induces chromatin condensation in cTVT cells. **(A)** Representative DAPI-stained fluorescence micrographs showing nuclear morphological changes following 24 h exposure to LD_50_ and LD_100_ concentrations of AgNPs-G (20× magnification, scale bar = 100 μm). **(B)** Quantification of chromatin condensation demonstrates a significant increase after treatment. Data are presented as mean ± SD. Statistical significance (****p ≤ 0.0001) determined with Dunnet’s post hoc test using one-way ANOVA.

**Figure 7:**
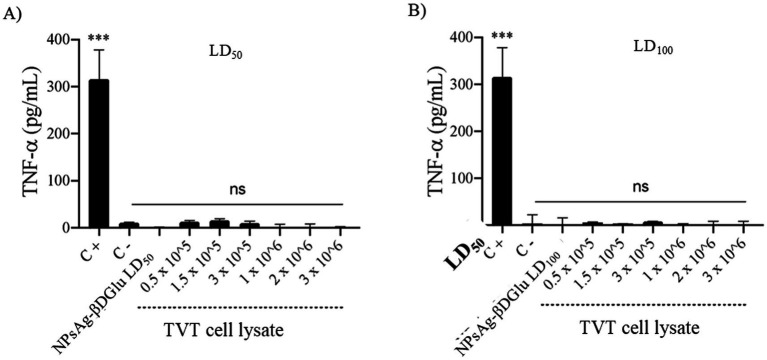
TNF-α levels in canine blood following exposure to AgNPs-G–treated cTVT cell lysates. Plasma TNF-α was measured after 24 h incubation of canine blood with increasing concentrations of cTVT cell lysates treated with AgNPs-G at LD_50_ **(A)** and LD_100_ **(B)**. Data are presented as mean ± SD (*n* = 3). Statistical significance (***p ≤ 0.001) determined with Dunnet’s post hoc test using one-way ANOVA.

**Figure 8:**
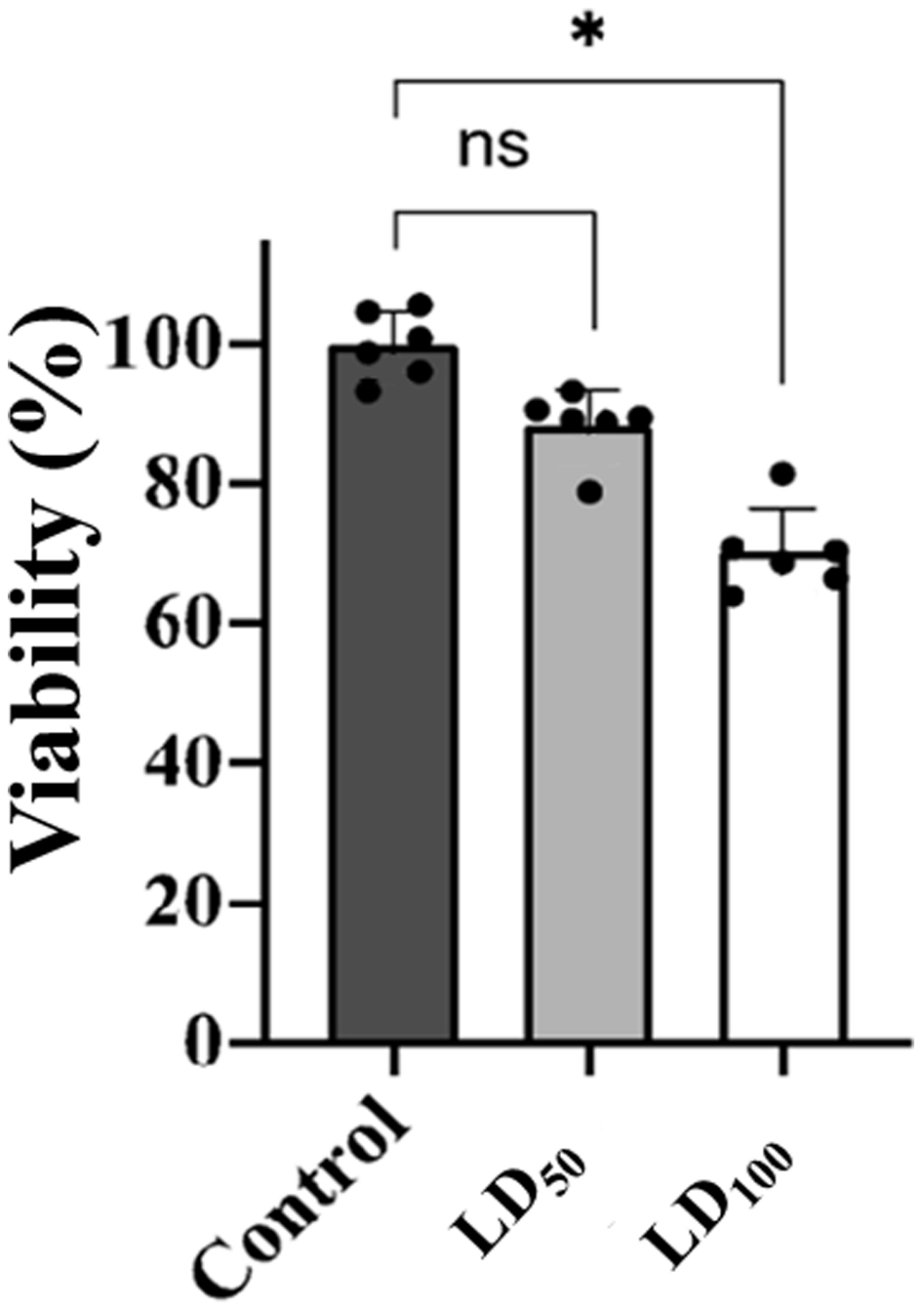
Effect of AgNPs-G on canine PBMC viability. PBMC viability was assessed after 24 h exposure to LD_50_ and LD_100_ concentrations of AgNPs-G. A significant reduction in cell viability was observed at the LD_100_ concentration. Data are presented as mean ± SD (*n* = 6). Statistical significance (*p ≤ 0.05) was determined using Dunnett’s post hoc test using one-way ANOVA. No significance (n.s.).

**Figure 9:**
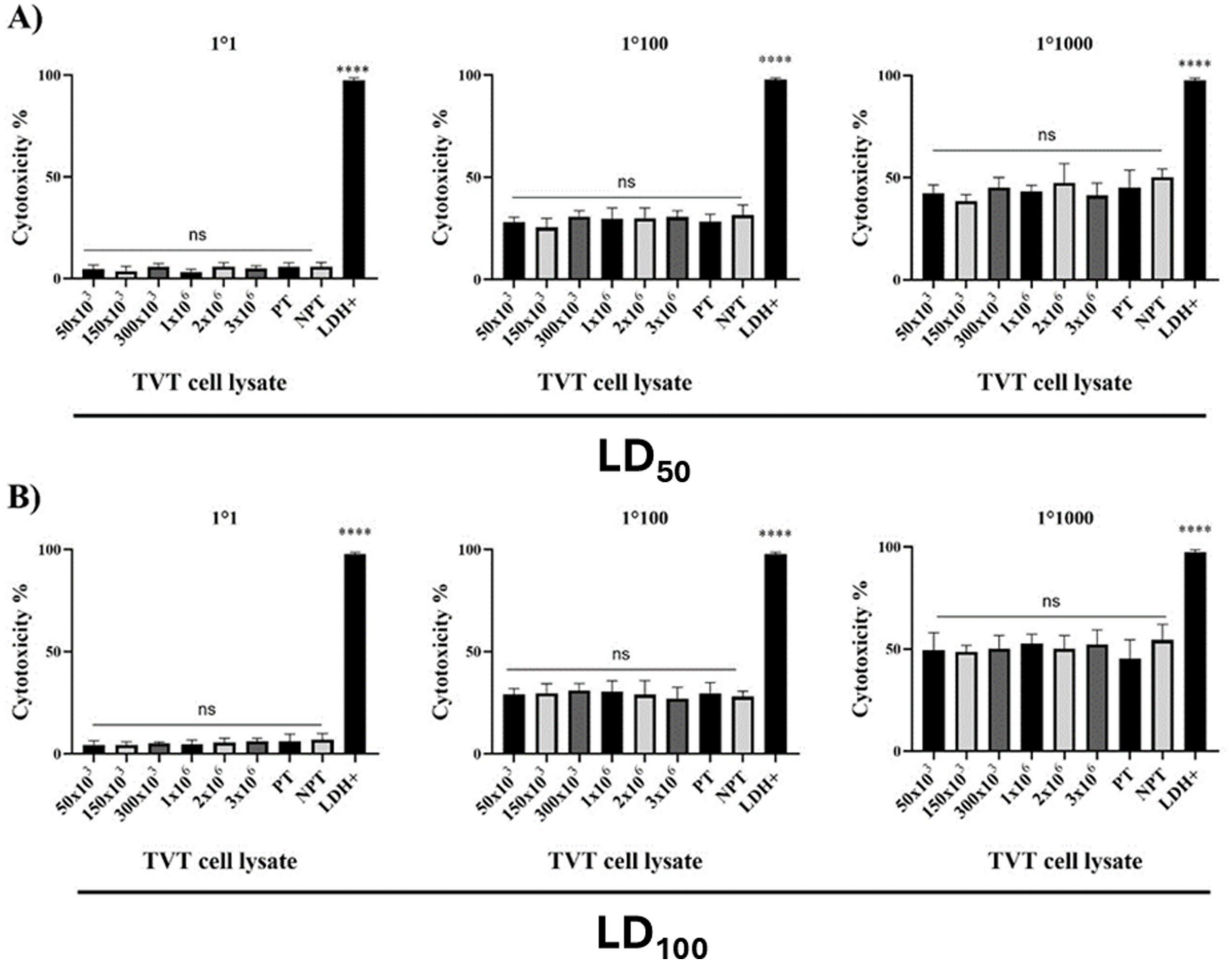
Cytotoxic response of PBMCs sensitized with AgNPs-G–treated cTVT cell lysates against cTVT cells. PBMC-mediated cytotoxicity was evaluated at different effector-to-target ratios following sensitization with cTVT cell lysates treated with AgNPs-G at LD_50_ **(A)** and LD_100_ **(B)**. No significant differences were observed among lysate-treated groups across the evaluated ratios, while the positive control (LDH^+^) showed maximal cytotoxic activity. Data are presented as mean ± SD (*n* = 3). Statistical significance (****p ≤ 0.0001) was determined using Dunnett’s post hoc test using one-way ANOVA.

All experimental data are presented as the mean ± standard deviation (SD). For *in vitro* assays (Figures 1–6), *n* = 3 represents three independent biological experiments performed with different cell preparations. For ex vivo assays (Figures 7–9), *n* = 3 represents independent biological samples obtained from three different canine donors. Technical triplicates were averaged to obtain a single value for each biological replicate.

The experiments were performed in triplicate. Statistical analysis was performed using one-way ANOVA followed by Dunnett’s/Tukey’s post-hoc tests (*p* < 0.05). All analyses were conducted using GraphPad Software version 6 (GraphPad Software, Inc., United States).

## 3 Results

### 3.1 Characterization of nanoparticles

**Figure 10:**
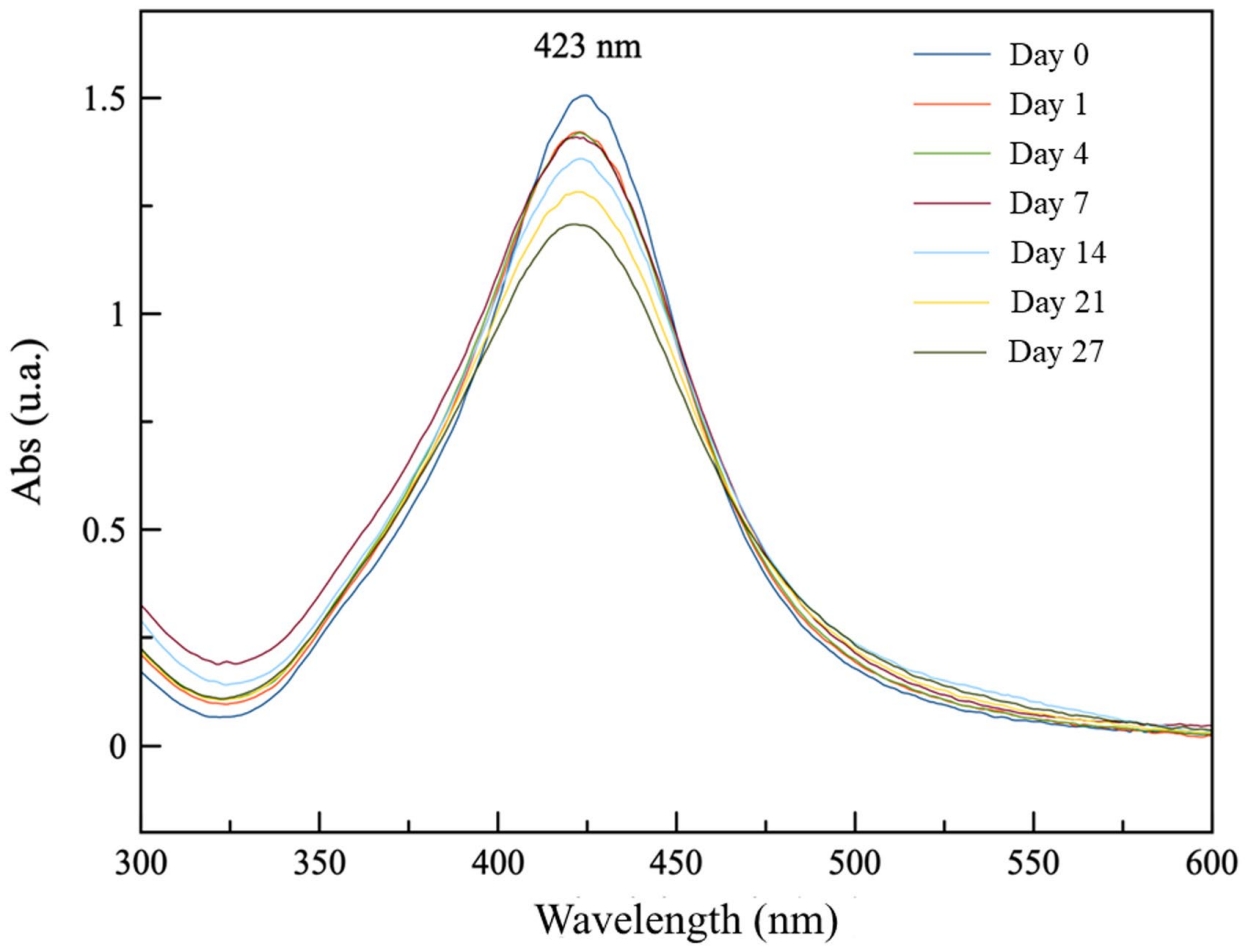
UV–vis spectra of AgNPs-G over a 27-day period.

UV–vis spectroscopy revealed a characteristic surface plasmon resonance peak of silver nanoparticles with a maximum absorbance at 423 nm, which remained stable throughout the evaluated period of 27 days (Figure 10). The UV–Vis spectrum showed a maximum absorbance peak at 423 nm, which is characteristic of spherical silver nanoparticles and consistent with our previous characterization of AgNPs-β-D-glucose (10). Dynamic light scattering (DLS) analysis showed that AgNPs-β-D-glucose had an average hydrodynamic diameter of 53.2 nm with a polydispersity index (PDI) of 0.21, indicating a narrow size distribution (Table 2). In addition, zeta potential measurements demonstrated that AgNPs-β-D-glucose possessed a surface charge of −24.8 mV, suggesting good colloidal stability.

**Table 2:** Physico-chemical characterization of AgNPs-G.

Day	Size	PDI	ζ potential
0	50.6	0.266	−33.6
1	57.33	0.233	−18.5
4	53.2	0.217	−24.8
7	57.14	0.247	−26.7
14	56.9	0.295	−24.4
21	54.63	0.216	−27
27	56.15	0.24	−24

### 3.2 Effects of the nanoparticles in the viability, ROS/RNS production and mitochondrial stress in cTVT cells

AgNPs-β-D-glucose significantly decreased cTVT cell viability in a dose-dependent manner (*p* ≤ 0.05). Based on the dose–response curve, the LD_50_ of AgNPs-β-D-glucose was determined to be 40 μM (Figure 1). cTVT cells treated with the LD_50_ of AgNPs-β-D-glucose exhibited a significant increase in ROS/RNS production compared with untreated cells (*p* ≤ 0.05), indicating enhanced oxidative stress following nanoparticle exposure (Figure 2). Additionally, GAPDH expression was significantly increased in cTVT cells treated with the LD_50_ of AgNPs-G for 24 h compared with untreated controls (*p* ≤ 0.01) (Figure 3). The elevated GAPDH levels suggest the induction of mitochondrial stress in response to AgNPs-β-D-glucose treatment.

### 3.3 Evaluation of cell death

Caspase-3 expression was significantly increased in cTVT cells treated with the LD_50_ of AgNPs-β-D-glucose for 24 h compared with untreated cells (*p* ≤ 0.01), indicating activation of the apoptotic pathway (Figure 4). Morphological changes associated with cell death were further evaluated using acridine orange/ethidium bromide (AO/EB) double staining and fluorescence microscopy. Viable cells exhibited uniform green fluorescence, apoptotic cells showed yellow fluorescence, and necrotic cells displayed red fluorescence (Figure 5A). Quantitative analysis revealed a significant increase in the percentage of apoptotic cells with increasing lethal doses of AgNPs-β-D-glucose (*p* ≤ 0.0001) (Figure 5B). In addition, DAPI staining demonstrated a significant increase in the proportion of apoptotic cells in cultures treated with the LD_50_ and LD_100_ of AgNPs-β-D-glucose compared with untreated controls (*p* ≤ 0.0001). Apoptotic cells were identified by intensified blue fluorescence and the presence of highly condensed nuclei, a hallmark of apoptosis (Figures 6A,B).

### 3.4 Evaluation of immunogenicity

The mean concentration of TNF-α in canine serum following exposure to cellular lysates (0.5 × 10^5^, 1.5 × 10^5^, 3 × 10^5^, 1 × 10^6^, 2 × 10^6^ and 3 × 10^6^ cells) generated from cTVT cells treated with AgNPs-G at LD_50_ and LD_100_ did not differ significantly from the negative control (*p* ≥ 0.001) (Figure 7).

Cell viability in whole blood samples treated with AgNPs-β-D-glucose (LD_50_ and LD_100_, corresponding to cTVT cells) was evaluated using the rezasurin assay. No significant reduction in viability was observed compared with untreated controls (*p* ≥ 0.05), with viability values remaining above 60% across all treated groups (Figure 8). These results indicate that peripheral blood cells remained viable and metabolically active following exposure. To assess whether AgNPs-β-D-glucose induced tumor cell death elicited an immunogenic response, peripheral blood mononuclear cells (PBMCs) were sensitized with lysates derived from AgNPs-β-D-glucose treated cTVT cells and subsequently co-cultured with cTVT cells. Cytotoxic activity was evaluated by measuring lactate dehydrogenase (LDH) release. No significant differences in LDH levels were detected between PBMCs sensitized with cTVT cells lysates (0.5 × 10^5^ to 3 × 10^6^ cells) generated using AgNPs-β-D-glucose at LD_50_ or LD_100_ and non-sensitized PBMC controls (*p* ≥ 0.0001) (Figure 9).

## 4 Discussion

cTVT is a disease with a substantial impact on canine health worldwide (6), highlighting the need to develop novel, evidence-based therapeutic strategies for its management. The present study provides the first evidence of the *in-vitro* effects of the AgNPs-β-D-glucose on cTVT cells.

The synthesized formulation of AgNPs-β-D-glucose employed, revealed a surface plasmon resonance peak with a maximum absorbance at 423 nm obtained thorough physicochemical characterization UV–Vis spectroscopy, indicating that absorption bands between 390 and 500 nm are characteristic of silver nanoparticles (16, 17). In the synthesis of AgNPs-G, NaOH was utilized to adjust the pH reaction. The presence of hydroxyl ions (OH) is critical to catalyze the deprotonation of β-D-glucose, which significantly increases its reducing potential. This condition facilitates the rapid reduction of silver ions (Ag+) to metallic silver (Ag^0^), ensuring better control over the nucleation process and resulting in the formation of stable, monodisperse nanoparticles. The zeta potential of AgNPs-G was −24.8 mV, indicating good colloidal stability that remained for 27 days and a low polydispersity index. This stability can be explained by electrostatic repulsion between nanoparticles, as values greater than ±5 mV progressively increase interparticle repulsion, as previously reported for maltose-stabilized silver nanoparticles with a zeta potential of −30 mV (18). This similarity may be attributed to the comparable chemical structures of β-D-glucose and maltose.

Functionally, AgNPs-β-D-glucose induced a significant reduction in the viability of the cTVT cell line after 24 h of exposure. These findings partially correlate with those reported by Panzarini et al. (19), who demonstrated a cytotoxic effect of glucose-coated silver nanoparticles (30 nm) on HeLa cells after 48 h of treatment. In parallel, a marked increase in oxidative stress was observed in AgNPs-β-D-glucose—treated cTVT cells. Oxidative stress has been widely associated with nanoparticle-induced cell death (20), and the presence of glucose in the nanoparticle system may further modulate this effect. Indeed, Vergallo et al. (21) reported that glucose and fructose can modify cellular responses in terms of viability, ROS generation, and induction of distinct cell death pathways in HeLa cells.

In agreement with the observed oxidative stress, AgNPs-G treatment led to a significant increase in GAPDH expression. Similar findings were reported by Davoudi et al. (13) in MCF-7 breast cancer cells treated with silver nanoparticles, where increased oxidative stress was associated with upregulation of GAPDH expression. Beyond its glycolytic role in converting glyceraldehyde-3-phosphate to 1,3-bisphosphoglycerate, GAPDH has been implicated in the activation of downstream molecular targets and the induction of apoptosis (22).

Consistent with this mechanism, caspase-3 expression was significantly increased following exposure to AgNPs-β-D-glucose, supporting the activation of apoptotic pathways. In the present study, approximately 50% of cTVT cells were positive for caspase-3 after nanoparticle treatment. Comparable results were reported by (23), who observed a 25% increase in caspase-3 expression in HT22 cells treated with silver nanoparticles; differences between studies may be attributed to variations in nanoparticle concentration and cell type.

The involvement of apoptosis was further confirmed by fluorescence microscopy. DAPI staining revealed an increased proportion of cells exhibiting nuclear condensation, a hallmark of apoptosis (24). Additionally, AO/EB double staining demonstrated a higher percentage of apoptotic cells, characterized by yellow fluorescence, whereas viable cells displayed green fluorescence and necrotic cells exhibited red fluorescence, as described by Tsangaris and Tzortzatou-Stathopoulou (25). These findings are consistent with previous reports showing a concentration-dependent increase in apoptotic cell populations following silver nanoparticle exposure in various cancer cell lines (26, 27). Collectively, these results indicate that AgNPs-β-D-glucose induce apoptotic cell death in cTVT cells. The induction of apoptosis by AgNPs-G in cTVT cells appears to be mediated by the orchestration of oxidative stress and the intrinsic apoptotic pathway. Our results demonstrated a significant increase in intracellular ROS and superoxide ion levels (Figure 2), which act as primary triggers for mitochondrial dysfunction. Silver nanoparticles are known to disrupt the mitochondrial membrane, leading to a loss of mitochondrial membrane potential and the subsequent release of pro-apoptotic factors into the cytosol (28, 29). In this context, Caspase-3 serves as the executioner protease, responsible for the systematic cleavage of key structural proteins and the activation of endonucleases, which directly correlates with the hallmark nuclear fragmentation and chromatin condensation observed in the DAPI and AO/EB assays (30). Also, apoptotic cell death can, under certain conditions, elicit an immune response through the exposure of damage-associated molecular patterns such as calreticulin, which can activate immune cells (31). Therefore, evaluating whether AgNPs-β-D-glucose—induced cell death is immunogenic is crucial for the potential development of nanoparticle-based immunotherapeutic strategies. One indicator of immune activation is the production of TNF-α, a cytokine involved in immune stimulation and antigen presentation (32). However, our results demonstrated that AgNPs-β-D-glucose treatment did not induce TNF-α production in canine blood samples. These findings are consistent with those reported by Haase et al. (33), who observed no activation of the TNF-α promoter in immune cells exposed to silver nanoparticles.

Furthermore, stimulation of PBMCs with cTVT cell lysates derived from AgNPs-β-D-glucose –treated cells did not induce TNF-α production. Although TNF-α plays a central role in immune activation (32), the absence of its induction suggests that AgNPs-β-D-glucose—induced cell death does not promote a strong immunogenic response under the conditions evaluated. To fully assess immune activation, future studies should include a broader panel of cytokines and immune activation markers. Additionally, although some studies have reported TNF-α production in peripheral blood cells treated with silver nanoparticles (34), our findings indicate that AgNPs-β-D-glucose did not exert sufficient immunostimulatory activity in this model.

Consistently, LDH release assays showed no increase in cytotoxic activity by PBMCs sensitized with cTVT lysates derived from AgNPs-G treatment, indicating impaired immune-mediated tumor cell elimination. Similar observations were reported by García-García et al. (35), who demonstrated that cell debris generated by silver nanoparticle–induced cell death failed to elicit an immune response. Together, these results suggest that AgNPs-β-D-glucose induce a non-immunogenic form of apoptotic cell death in cTVT cells. Nevertheless, these findings are limited to *in vitro* conditions and should be validated *in vivo*.

Although authors showed no effect of the nanoparticles on the biology of immune cells, their test on a normal, non-tumor cell line should be recommended.

Future studies should explore the combined use of AgNPs-β-D-glucose with vincristine, the standard chemotherapeutic agent for cTVT, to evaluate potential synergistic effects. While vincristine disrupts microtubule dynamics during mitosis, AgNPs-β-D-glucose may act through alternative mechanisms such as oxidative stress induction and membrane damage, potentially enhancing therapeutic efficacy. Moreover, β-D-glucose may contribute not only as a reducing agent but also to nanoparticle stabilization and metabolic targeting, given the high demand of cancer cells. In conclusion, silver nanoparticles with an average size of 53.2 nm induced apoptosis-dependent cell death in the cTVT cell line, likely mediated through increased ROS production and the upregulation of GAPDH and caspase-3. Nevertheless, further studies are required to establish optimal dosing, therapeutic efficacy, and safety before considering their application in the clinical treatment of cTVT.

## Data Availability

The original contributions presented in the study are included in the article/supplementary material, further inquiries can be directed to the corresponding authors.

## References

[1] Abeka YT. Review on canine transmissible venereal tumor (CTVT). Cancer Ther Oncol Internat J. (2019) 14:555895. doi: 10.19080/CTOIJ.2019.14.555895

[2] Ní Leathlobhair M Lenski RE. Population genetics of clonally transmissible cancers. Nature Ecol Evolut. (2022) 6:1077–89. doi: 10.1038/s41559-022-01790-3, 35879542

[3] Nak D Nak Y Cangul IT Tuna B. A clinico-pathological study on the effect of vincristine on transmissible venereal tumour in dogs. J Veterinary Med Ser A. (2005) 52:366–70. doi: 10.1111/j.1439-0442.2005.00743.x, 16109105

[4] Lakde CK Bind AA Sahatpure SK. Diagnosis and clinical treatment of transmissible venereal tumor in canines. Int J Curr Microbiol App Sci. (2020) 9:179–82. doi: 10.20546/ijcmas.2020.909.022

[5] Liu CC Wang YS Lin CY Chuang TF Liao KW Chi KH . Transient downregulation of monocyte-derived dendritic-cell differentiation, function, and survival during tumoral progression and regression in an in vivo canine model of transmissible venereal tumor. Cancer Immunol Immunother. (2008) 57:479–91. doi: 10.1007/s00262-007-0386-0, 17710396 PMC11030039

[6] Luo M Wang YM Zhao FK Luo Y. Recent advances in nanomaterial-mediated cell death for cancer therapy. Adv Healthc Mater. (2025) 14:e2402697. doi: 10.1002/adhm.202402697, 39498722

[7] Khan I Saeed K Khan I. Nanoparticles: properties, applications and toxicities. Arab J Chem. (2019) 12:908–31. doi: 10.1016/j.arabjc.2017.05.011

[8] Kovács D Igaz N Gopisetty MK Kiricsi M. Cancer therapy by silver nanoparticles: fiction or reality? Int J Mol Sci. (2022) 23:839. doi: 10.3390/ijms23020839, 35055024 PMC8777983

[9] Vergallo C Panzarini E Carata E Ahmadi M Mariano S Tenuzzo BA . Cytotoxicity of β-D-glucose/sucrose-coated silver nanoparticles depends on cell type, nanoparticle concentration and time of incubation. AIP Conf Proc. (2016) 1749:020012. doi: 10.1063/1.4954495

[10] Félix-Piña P Franco-Molina MA Zárate-Triviño DG García-Coronado PL Zapata-Benavides P Rodríguez-Padilla C. Antitumoral and immunogenic capacity of β-D-glucose-reduced silver nanoparticles in breast cancer. Int J Mol Sci. (2023) 24:8485. doi: 10.3390/ijms24108485, 37239831 PMC10217844

[11] Zayas YR Molina MAF Guerra RT Padilla CR. Evaluation of a canine transmissible venereal tumour cell line with tumour immunity capacity but without tumorigenic property. J Vet Res. (2019) 63:225–33. doi: 10.2478/jvetres-2019-0024, 31276062 PMC6598177

[12] Kot Y Prokopiuk V Klochkov V Tryfonyuk L Maksimchuk P Aslanov A . Mn_3_O_4_ nanocrystal-induced eryptosis features Ca^2+^ overload, ROS and RNS accumulation, calpain activation, recruitment of caspases, and changes in the lipid order of cell membranes. Int J Mol Sci. (2025) 26:3284. doi: 10.3390/ijms26073284, 40244142 PMC11989249

[13] Davoudi M Moradi-Sardareh H Emamgholipour S Nabatchian F Paknejad M. The possible effect of silver nanoparticles on glyceraldehyde-3-phosphate dehydrogenase activity and formation of amyloid-like aggregates in MCF-7 cell line. IUBMB Life. (2020) 72:2214–24. doi: 10.1002/iub.2362, 32819028

[14] Cho HW Seo K Chun JL Jeon J Kim CH Lim S . Effects of resistant starch on anti-obesity status and nutrient digestibility in dogs. J Anim Sci Technol. (2023) 65:550–61. doi: 10.5187/jast.2023.e11, 37332283 PMC10271923

[15] Bautista CMS Amante BM Ruamero EC Jr. Determination of toxicity and hypoglycemic effect in alloxan-induced diabetic mice of *Manihot esculenta* Crantz aqueous crude leaf extract and its fractions. Acta Med Philipp. (2025) 59:65–74. doi: 10.47895/amp.vi0.7624, 40438488 PMC12106103

[16] Dawadi S Katuwal S Gupta A Lamichhane U Thapa R Jaisi S . Current research on silver nanoparticles: synthesis, characterization, and applications. J Nanomater. (2021) 2021:6687290. doi: 10.1155/2021/6687290

[17] Pechyen C Tangnorawich B Toommee S Marks R Parcharoen Y. Green synthesis of metal nanoparticles, characterization, and biosensing applications. Sensors Int. (2024) 5:100287. doi: 10.1016/j.sintl.2024.100287

[18] Katsumiti A Gilliland D Arostegui I Cajaraville MP. Mechanisms of toxicity of ag nanoparticles in comparison to bulk and ionic ag on mussel hemocytes and gill cells. PLoS One. (2015) 10:e0129039. doi: 10.1371/journal.pone.0129039, 26061169 PMC4465040

[19] Panzarini E Mariano S Vergallo C Carata E Fimia GM Mura F . Glucose-capped silver nanoparticles induce cell cycle arrest in HeLa cells. Toxicol In Vitro. (2017) 41:64–74. doi: 10.1016/j.tiv.2017.02.014, 28223142

[20] Dini L Panzarini E Serra A Buccolieri A Manno D. Synthesis and in vitro cytotoxicity of glycans-capped silver nanoparticles. Nanomater Nanotechnol. (2011) 1:10. doi: 10.5772/50952, 41762079

[21] Vergallo C Panzarini E Izzo D Carata E Mariano S Buccolieri A . Cytotoxicity of β-D-glucose-coated silver nanoparticles on human lymphocytes. AIP Conf Proc. (2014) 1603:78–85. doi: 10.1063/1.4883045

[22] Zhang JY Zhang F Hong CQ Giuliano AE Cui XJ Zhou GJ . Critical protein GAPDH and its regulatory mechanisms in cancer cells. Cancer Biol Med. (2015) 12:10–22. doi: 10.7497/j.issn.2095-3941.2014.0019, 25859407 PMC4383849

[23] Ma W Jing L Valladares A Mehta SL Wang Z Li PA . Silver nanoparticle exposure induced mitochondrial stress, caspase-3 activation and cell death: amelioration by sodium selenite. Int J Biol Sci. (2015) 11:860–7. doi: 10.7150/ijbs.12059, 26157341 PMC4495404

[24] Chazotte B. Labeling nuclear DNA using DAPI. Cold Spring Harb Protoc. (2011) 2011:pdb.prot5556. doi: 10.1101/pdb.prot5556, 21205856

[25] Tsangaris GT Tzortzatou-Stathopoulou F. Development of a quantitative method for the study of apoptosis in peripheral blood. In Vivo. (1996) 10:435–43. 8839791

[26] Baharara J Namvar F Ramezani T Mousavi M Mohamad R. Silver nanoparticles biosynthesized using *Achillea biebersteinii* flower extract: apoptosis induction in MCF-7 cells via caspase activation and regulation of Bax and Bcl-2 gene expression. Molecules. (2015) 20:2693–706. doi: 10.3390/molecules20022693, 25665064 PMC6272258

[27] Gopinath P Gogoi SK Chattopadhyay A Ghosh SS. Implications of silver nanoparticle-induced cell apoptosis for in vitro gene therapy. Nanotechnology. (2008) 19:075104. doi: 10.1088/0957-4484/19/7/075104, 21817629

[28] Hsin YH Chen CF Huang S Shih TS Lai PS Chueh PJ. The apoptotic effect of nanosilver is mediated by a ROS- and JNK-dependent mechanism involving the mitochondrial pathway in NIH3T3 cells. Toxicol Lett. (2008) 179:130–9. doi: 10.1016/j.toxlet.2008.04.015, 18547751

[29] Piao MJ Kang KA Lee IK Kim HS Kim S Choi JY . Silver nanoparticles induce oxidative cell damage in human liver cells through inhibition of reduced glutathione and induction of mitochondria-involved apoptosis. Toxicol Lett. (2011) 201:92–100. doi: 10.1016/j.toxlet.2010.12.010, 21182908

[30] AshaRani PV Low Kah Mun G Hande MP Valiyaveettil S. Cytotoxicity and genotoxicity of silver nanoparticles in human cells. ACS Nano. (2009) 3:279–90. doi: 10.1021/nn800596w, 19236062

[31] Kono H Rock KL. How dying cells alert the immune system to danger. Nat Rev Immunol. (2008) 8:279–89. doi: 10.1038/nri2215, 18340345 PMC2763408

[32] Parameswaran N Patial S. Tumor necrosis factor-α signaling in macrophages. Crit Rev Eukaryot Gene Expr. (2010) 20:87–103. doi: 10.1615/CritRevEukaryotGeneExpr.v20.i2.10, 21133840 PMC3066460

[33] Haase H Fahmi A Mahltig B. Impact of silver nanoparticles and silver ions on innate immune cells. J Biomed Nanotechnol. (2014) 10:1146–56. doi: 10.1166/jbn.2014.1784, 24749409

[34] Park MV Neigh AM Vermeulen JP de la Fonteyne LJ Verharen HW Briedé JJ . The effect of particle size on the cytotoxicity, inflammation, developmental toxicity and genotoxicity of silver nanoparticles. Biomaterials. (2011) 32:9810–7. doi: 10.1016/j.biomaterials.2011.08.085, 21944826

[35] García-García MR Casares N Martínez-Pérez LA Juárez-Curiel E de Jesús-Hernández AA Bogdanchikova N . Silver nanoparticles induce a non-immunogenic tumor cell death. J Immunotoxicol. (2023) 20:2175078. doi: 10.1080/1547691X.2023.2175078, 36773297

[36] Franco Molina MA Reding Hernández D García Coronado PL Kawas JR Zárate Triviño DG Hernández Martínez SP . Antitumor efficacy of silver nanoparticles reduced with β-D-glucose as neoadjuvant therapy to prevent tumor relapse in a mouse model of breast cancer. Front. Pharmacol. (2024) 14:1332439. doi: 10.3389/fphar.2023.1332439 38333224 PMC10851876

